# The Effect of Calcium Peroxide on the Phenol Oxidase and Acid Phosphatase Activity and Removal of Fluoranthene from Soil

**DOI:** 10.1007/s11270-015-2632-y

**Published:** 2015-10-07

**Authors:** Anna Małachowska-Jutsz, Magdalena Niesler

**Affiliations:** Environmental Biotechnology Department, Silesian University of Technology, Akademicka 2A, 44-100 Gliwice, Poland

**Keywords:** Fluoranthene, AOPs, Phenol oxidase, Acid phosphatase, Soil decontamination

## Abstract

A study has been conducted to enhance fluoranthene degradation by combining biodegradation with hydrogen peroxide oxidation, as a chemical oxidant calcium peroxide has been used. The impacts of addition of calcium peroxide on microbial activity (phenol oxidase and acid phosphatase) as well as fluoranthene removal efficiency were investigated. It was observed that in the presence of calcium peroxide, the removal efficiency of fluoranthene on day 30 of the experiment was threefold higher as compared to a reference sample. It was found that the activity of phenol oxidase was stimulated on days 1, 7, and 14, by the presence of fluoranthene, whereas stimulation of the acid phosphatase activity in the samples of soil contaminated by fluoranthene was observed only after 14 days of the experiment. This may indicate that the induction period for this enzyme is longer compared with the induction period for phenol oxidase. The inhibition of the activity of both enzymes was observed in the presence of calcium peroxide.

## Introduction

Polycyclic aromatic hydrocarbons (PAHs) are commonly occurring contaminants. Their poor solubility in water causes them to be highly absorbed by soil and bottom sediments. This makes their accessibility limited for microorganisms responsible for the biodegradation process. Fluoranthene which is one of 16 priority pollutant PAHs according to Environmental Protection Agency consists of naphthalene and benzene unit connected by a five-membered ring. PAHs having four or more aromatic rings, e.g., fluoranthene, are more recalcitrant to microbial degradation. Fluoranthene is the most common and persistant PAHs pollutants and is therefore frequently chosen as a model compound for ex situ biodegradation studies (Sepic et al. 2003). When the efficacy of biodegradation is insufficient and the rate of natural degradation of the contaminants is too low, then it is necessary to use additional solutions. Chemical oxidation is a promising degradation method for an extensive variety of persistent compounds, e.g., PAHs. The recent studies demonstrated that chemical oxidation can coexist with aerobic metabolism in soils and that combination is more effective solution compared to natural bioremediation processes. In Advanced Oxidation Processes (AOPs), highly reactive free radicals are formed, especially hydroxyl radicals that have a high redox potential (2.8 V) and the capability to oxidize practically any organic compound (Biń and Zieliński 2000; Liu et al. 2014). Currently, research is being done to find the possibility of using other reagents in the processes of advanced oxidation. Compounds like peroxides are of high interest. Peroxides are a group of compounds that contain a peroxide group in its structure. During the degradation of these compounds, H_2_O_2_ (1, 2, 3) is produced, which is the source of oxygen for microorganisms taking active part in the biodegradation of contaminants while at the same time being the source of free radicals capable of oxidizing organic contaminants.

Calcium peroxide naturally decomposes very slowly to form calcium hydroxide and oxygen.

Depending upon the environment, the decomposition proceeds according to the reactions below:

1 $$ 2{\mathrm{CaO}}_2 + 2{\mathrm{H}}_2\mathrm{O}\ \to\ 2\mathrm{C}\mathrm{a}{\left(\mathrm{O}\mathrm{H}\right)}_2 + {\mathrm{O}}_2 $$

or

2 $$ {\mathrm{CaO}}_2 + 2{\mathrm{H}}_2\mathrm{O}\ \to\ \mathrm{C}\mathrm{a}{\left(\mathrm{O}\mathrm{H}\right)}_2 + {\mathrm{H}}_2{\mathrm{O}}_2 $$

3 $$ 2{\mathrm{H}}_2{\mathrm{O}}_2\to\ 2{\mathrm{H}}_2\mathrm{O} + {\mathrm{O}}_2 $$

The application of calcium peroxide has been found to be one of the most efficient for removal petroleum hydrocarbons, polycyclic aromatic hydrocarbons, tetrachloroethylene, and 2,4,6-trinitrotoluene from soil (Cassidy et al. 2008; Goi et al. 2011; Khodaweisi et al. 2011; Bianchi-Mosquera et al. 1994; Arienzo 2000; Hanh et al. 2005). Low solubility of calcium peroxide allows continuous release of hydrogen and oxygen peroxide for a long period of time (Goi et al. 2011). Moreover, the addition of calcium peroxide allows decomposition of contaminations in a wide pH range (Arienzo 2000). In order to state the full success of bioremediation, one cannot take into account only the degradation (full or partial) of the contaminant. It often occurs that during the degradation of PAHs, substances that are more toxic than the initial ones appear in the ecosystem. The progress of the bioremediation process of soils contaminated with hydrocarbons is usually monitored with the use of conventional methods of chemical analysis, which however do not evaluate the actual influence of these contaminants on the biotic elements of the environment. Based on literature review, it is known that in a simplified but reliable way, the process of bioremediation of soils contaminated with hydrocarbons can be tracked using, among others, activity of polyphenol oxidase, acid phosphatase, and dehydrogenase (Chaudhary et al. 2012). Research of the activity of the enzymes of soil microorganisms is widely used to determine biodiversity and the quality of soil (Chaudhary et al. 2012; Quasenian et al. 2012; Małachowska-Jutsz and Miksch 2010). The microbiological activity of soil depends, inter alia, on the type of contamination and the concentration of the analyzed compound (Chaudhary et al. 2012). The enzymes taking active part in the degradation of polycyclic aromatic hydrocarbons, including fluoranthene, are, inter alia, oxygenase, dehydrogenase, ligninolytic enzymes (lignin peroxidase and peroxidase dependent on Mn), and phenol oxidase (Haritash and Kauskik 2009).

In these research, the process of remediation of contaminated soil was stimulated by adding calcium peroxide which been used as activator for fluoranthene degradation on laboratory scale. Two concentrations of calcium peroxide selected in previous experiment (Małachowska-Jutsz et al. 2014a, b) were examined. The influence of dose calcium peroxide on phenol oxidase and acid phosphatase as well as fluoranthene removal efficiency was investigated. Determining the activity of acid phosphatase and polyphenol oxidase was selected for the research because polyphenol oxidase may give indications on the oxidative potential of soil (Gianfreda et al. 2005), while acid phosphatase is actively involved in phosphorus metabolism in soil and is widely determined in research of the effects of various pollutants on soil microflora. The proposed approach is the combination of biological mediation methods with chemical processes, which allow to increase the removal of hydrocarbons efficiency and the reduction of processing time.

## Materials and Methods

The soil (loamy sand) was collected from the 0–20-cm layer of uncontaminated land Kotlarnia. The soil was dried at room temperature (22–25 °C) for 2 weeks and passed through a sieve with a mesh size of 2 mm, in order to homogenize it. An analysis of basic physicochemical parameters was performed in accordance to the methodology of Ostrowska et al. (1991) which consisted of analyzing pH, organic substance, organic carbon (Tiurin method), buffering (Arrhenius method), hydrolytic acidity (Kappen method), total nitrogen (Kjeldahl method), phosphorus (ISO 11263, 1994); the distribution of soil particle size was established by an aerometric method (PN-R-04032 1998). Several characteristics of the soil are presented in Table 1. The experiment and analysis were performed in triplicate.

**Table 1:** Physical-chemical parameters of soil

Parameter	Value (mean ± standard deviation)
pH_H2O_	6.33 ± 0.17
Organic matter	12.068 ± 0.38 g/kg
Organic carbon	7.0 ± 0.22 g/kg
Buffer capacity	0.24 ± 0.02 [B]
Hydrolytic acidity	0.525 ± 0.03 [cmol (+)/kg]
Total nitrogen	0.55 ± 0.022 g/kg
Phosphorus (P_2_O_5_)	0.018 ± 0.0027 g/kg
Sand	84.05 %
Silt	2.55 %
Clay	13.4 %

Ten kilograms of soil was added into each of six containers with a volume of 20 l (each). The research was conducted in the following combinations (Table 2). Calcium peroxide (CaO_2_), powder, 200 mesh (0.075 mm), purchased from Aldrich, was applied for that soil treatment. The contents of CaO_2_ in calcium peroxide were 76.4 ± 0.1 % (with 17.0 ± 0.1 % of available oxygen). The soil samples were mixed with calcium peroxide. Remediation was carried out for 30 days in 22–25 °C. In this time, the soil was systematically moistened used distillate water in order to maintain moisture of 60 % water whole capacity, and it was mixed. The soil in each of six containers was daily mixed. Samples for enzyme assay and fluoranthene concentration determination were collected in four replicates, after 1, 7, 14, and 30 days of treatment.

**Table 2:** Experimental design

Sample	Calcium peroxide (mg/kg soil)	Fluoranthene (mg/kg soil)
1	0	0
2	0	1.5
3	120	0
4	240	0
5	120	1.5
6	240	1.5

### Determination of Fluoranthene Concentration

The soil samples were dried in a dryer in a temperature of 30 °C and then passed through a sieve with a mesh size of 2 mm. Extraction of fluoranthene from the soil was done using dichloromethane (CH_2_Cl_2_) in an automatic Soxtherm extractor (Gerhardt brand) in a temperature of 135 °C for 3 h. Fifty grams of dried and homogenized soil was used for the extraction. The extracts were quantitatively transferred to beakers with a volume of 25 ml and were concentrated in room temperature with a gentle stream of nitrogen to a volume of about 1 ml. The concentrated extracts were cleaned on glass columns diameter of 1 cm, filled with 1 g of Florisil (deactivated with 4 % of water). The fraction containing fluoranthene was washed out of the deposit by washing it twice with dichloromethane on order to obtain 10 ml of eluate. The eluate was concentrated in room temperature using a gentle stream of nitrogen to a volume of about 1 ml. An HP1050 liquid chromatography with a HP1046 fluorescence detector from Hewlett-Packard was used to determine the content of fluoranthene. A BAKERBOND PAH 16 Plus column with Baker precolumn was used. The dimensions of the column: length 250 mm, inner diameter 3 mm. It was filled with nonpolar phase C18 with a particle size of 5 μm.

### The Acid Phosphatase Activity Assay

The acid phosphatise activity was determined by the Tabatabai and Bremner method (Page 1982). One gram of soil and 0.25 ml of toluene were added into an Erlenmeyer flask and carefully mixed. The flask was tightly closed and left for 10 min. Afterward, 4 ml of MUB buffer with pH 6.5 and 1 ml of *p*-nitrophenyl phosphate sodium buffer solution were added. The flask was closed and mixed for a few seconds and then put into incubation in a temperature of 37 °C for 1 h. After the incubation, the reaction was interrupted by adding 1 ml of 0.5 M calcium chloride and 4 ml of 0.5 M sodium hydroxide. It was mixed for a few seconds, and then filtered through filter paper (Whatman no. 12) into a test tube. The absorbance of the filtrate was measured with a spectrophotometer at a wavelength of 400 nm. The concentration of *p*-nitrophenol expressed in milligrams per kilogram was calculated based on a calibration curve.

Activity of acid phosphatase expressed in $\frac{mg}{g\ast h}$ was calculated using the formula:

$$ \mathrm{Activity}=\frac{released\;p- nitrophenol(mg)}{soil\; sample(g)\ast incubation\; time(h)}\left[\frac{mg}{g\ast h}\right] $$

### The Phenol Oxidase Activity Assay

The phenol oxidase activity was determined by Bob Sinsabaugh method (Allison Lab Protocol 2008). Ten grams of soil and 50 ml of acetate buffer were added into 50-ml Erlenmeyer flask and stirred. The 2 ml of soil suspension was transferred into test tube. To the same test tube, 2 ml of 5 mM solution of L-DOPA was added. In parallel, a control sample was prepared which contained 2 ml of soil suspension and 2 ml of acetate buffer. The test sample was mixed thoroughly and incubated for 1 h at 20 °C and then centrifuged. The absorbance of the test sample supernatant and the control sample was measured at a wavelength of 460 nm. All the measurements were made in four replicates.

The phenol oxidase activity, expressed in $\frac{\upmu \mathrm{mol}}{g\ast h}$ was calculated according to the formula:

OD = absorbance of the sample − absorbance of the control sample

$$ \mathrm{Activity}=\frac{OD}{1,66\ast incubation\ast \left(\frac{dry\; soil\; sample(g)}{suspension(ml)}\right)}\left[\frac{\upmu \mathrm{mol}}{g\ast h}\right] $$

### Statistical Analysis and Enzyme Assay

Statistical analysis of the results included the calculation of the arithmetic mean and standard deviation. To verify the distribution normality, the Shapiro-Wilk test was applied. The significance of differences between individual samples was assessed using Student’s *t* test. Differences were considered statistically significant if *p* < 0.05. Statistical analysis was performed using Statistica 10 StatSoft, Inc., software.

Results of enzyme activity were calculated as percentage of the control sample as:

$$ \mathrm{A}=\frac{K-B}{K}\ast 100\%\left[\%\right] $$

where:

A: percentage of enzyme activity against the control sample [%]
K: mean enzyme activity in control sample
B: mean enzyme activity in test sample

## Results and Discussion

### Fluoranthene Removal in Soil

**Fig. 1:**
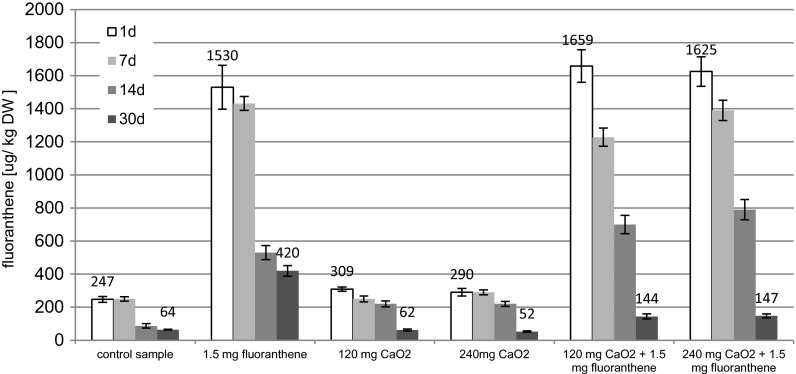
Changes of fluoranthene concentration in soil samples

The chemical structure of polycyclic aromatic hydrocarbons is one of the main factors, which have influence in their degradation rate. Hydrocarbons which contain two to three rings in the molecule are relatively easily biodegradable, and the decomposition rate is inversely proportional to the number of aromatic rings. In the case of hydrocarbons with four or more aromatic rings, the decomposition is very slow and often requires the presence of cometabolites. Crucial effect on the biodegradation of the compounds is the presence of microorganisms capable to degrade these substances. In the present study, it was found that control soil sample was contaminated with 247 μg/kg dry weight (DW) of fluoranthene. Many hydrocarbons, including fluoranthene, undergo degradation after a period of adaptation of microorganisms. At that time, they produce inductive enzymes. This process is influenced by, among others, the concentration of hydrocarbons as carbon sources: Too low limits or even prevents the production of these enzymes, and too high is toxic. In the research, it was found that in the samples into which 1.5 mg of fluoranthene/kg of soil was introduced, the biodegradation proceeded efficiently. After 30 days of self-cleaning process, the decreases of fluoranthene concentration in control sample and samples, to with 1.5 mg/kg fluorathene was added, were respectively 74.23 % (of 0.25 mg/kg) and 77 % (Fig. 1). In soils with calcium peroxide, higher fluoranthene concentration was determined (Fig. 1). This indicates the presence of microorganisms capable to degrade the hydrocarbon. The decomposition process is also affected by the soil moisture. Literature data indicate that PAHs are the most stable in air-dry soils (less than 1 % of water holding capacity (WHC)), and the biodegradation process runs most efficiently at a relative humidity of 60–65 % of WHC (Chiou 1989). This humidity was maintained throughout the experimental period, i.e., 30 days. In soils with low moisture sorption of PAHs is stronger than in the hydrated systems because organic compounds can be adsorbed not only by the soil organic matter but also by clay minerals (Maliszewska-Kordybach 1990). Another very important issue is the content of organic matter in the soil. Slightly loamy sand with low organic matter content (Table 1) in order to eliminate the influence of sorption process of fluorantede degradation was intentionally chosen. On the other hand, studies show that the microbial activity in soils was a critical factor governing the degradation of organic micro-pollutants (Małachowska-Jutsz et al. 2014a). The present study was conducted to analyze the effects of soil organic matter on the development of degradation potentials for polycyclic aromatic hydrocarbons (PAHs). Most of the degradation kinetics for PAHs by the indigenous microorganisms developed in soils can be fitted with the Logistic growth models. The microbial activities were relatively lower in the soils with the lowest and highest organic matter content, which were likely due to the nutrition limit and PAH sequestration. The microbial activities developed in humic acids (HA) were much higher than those developed in humic acids, which was demonstrated to be able to sequester organic pollutants stronger. The results suggested that the nutrition support and sequestration were the two major mechanisms that soil organic matter influenced the development of microbial PAHs degradation potentials (Yang et al. 2012).

The addition of calcium peroxide to the soil samples, to which fluoranthene in concentration of 1.5 mg/kg of soil was added, caused an increase in fluoranthene removal. After 30 days, the removal degree of fluoranthene, in soil samples with calcium peroxide in concentration of 120 mg/kg of soil and 240 mg/kg of soil, was respectively 90.11 and 90.2 % (Fig. 1). The same phenomena was observed by Arienzo (2000). The addition of low concentrations of calcium peroxide (0.2 %) to soils contaminated with PAHs does not lead to sterilization the soil and did not cause significant changes in the metabolic activity of microorganisms. However, the additional condition was to provide the microorganisms biogenic substances in an effective amount. Generally, a molar weight ratio of carbon to nitrogen to potassium to phosphorous of from 100:10:10:10 to 100:10:1:1 is employable, with a preferred ratio of from 100:10:5:5 to 100:10:1:1, and a most preferred ratio of from 100:10:4:4 to 100:10:1 :1 (patent The bioremediation of soils containing hydrocarbon using calcium peroxide - WO 1994029242 A1, 1994). In the present study, no biogenic substances were added.

pH is an important factor influencing the degradation of PAHs. The pH was monitored in the course of the study (Table 3). The addition of calcium peroxide caused the increase of pH value from 6.33 to 6.84 for 120 mg CaO_2_/kg and to 7.16 for 240 mg CaO_2_/kg of soil samples. Many studies have shown that optimal conditions for microbial degradation of hydrocarbons are pH 6.5–7.5 (Małachowska-Jutsz and Miksch 2010; Gan and Ng 2012). According to Pawar (2012) bacterial populations were greater at pH 7.5 which was highly correlated with soil adenosine triphosphate (ATP) levels. It was therefore evident that the greatest rates of PAHs degradation were associated with the greatest bacterial population. Soil enzyme activities in general were also greatest at pH 7.5.

**Table 3:** Changes of pH_H2O_

Sample	1 day	7 days	14 days	30 days
Control sample	6.33	6.31	6.23	6.27
1.5 mg fluoranthene	6.40	6.42	6.29	6.28
120 mg CaO_2_	6.84	6.72	6.78	6.79
240 mg CaO_2_	7.16	6.96	7.00	7.28
120 mg CaO_2_ + 1.5 mg fluoranthene	7.02	6.76	6.77	7.16
1240 mg CaO_2_ + 1.5 mg fluoranthene	7.28	6.75	7.02	7.38

### The Changes of Acid Phosphatase Activity During Remediation

Soil also contains a resource of available enzymes secreted from living cells of microorganisms and released into the environment in the lytic processes, which operate independently from parent cells, and their activity is controlled by the conditions in the environment (Brzezińska 2006; Dick 1997; Gianfreda et al. 2005).

Acid phosphomonoesterase, commonly referred to as acid phosphatase, is actively involved in the first stage of decomposition of organic matter in the soil environment; thereby, it is involved in the processes of mineralization of organic phosphorus. The activity of phosphatases in soil reflects the activity of enzymes associated with soil colloids and humic substances, free phosphatases in the soil solution, and phosphatases associated with live and dead cells of plants and microorganisms. The main source of phosphatases in soil is primarily soil microorganisms and plant roots and soil fauna.

**Fig. 2:**
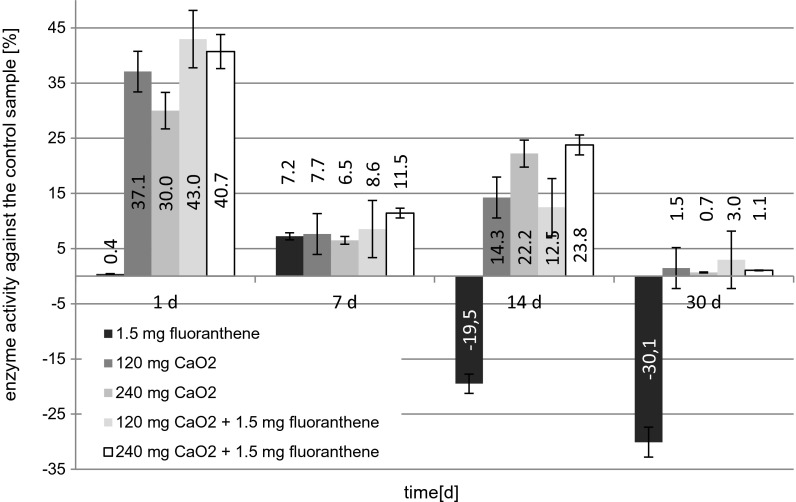
The effect of fluoranthene and calcium peroxide on the activity of acid phosphatase

The effect of soil modifications on the activity of acid phosphatase is shown in Fig. 2. The addition of fluoranthene on days 1 and 7 of the experiment resulted in slight decrease of acid phosphatase activity in comparison with a control sample. Stimulation of the enzyme activity was observed in the course of the process of the remediation of the soil contaminated with fluoranthene after 14 and 30 days. This may be indicative of the contribution of this enzyme to the biodegradation process of fluoranthene and also of relatively long period of induction of this enzyme by soil microorganisms (Borràs et al. 2010). On day 30 of the study, acid phosphatase activity in the samples contaminated with fluoranthene was on average about 30 % higher than in a reference sample. The highest percentage of activity decrease of acid phosphatase (37.08 %) was observed after day 1 of the experiment in the soil sample to which CaO_2_ was added at a concentration of 120 mg/kg soil after, while in the subsequent days of the experiment, the phosphatase activity increased. On day 30 of the experiment, acid phosphatase activity in the sample with the addition of calcium peroxide was almost the same as in the control. For the soil sample containing CaO_2_ (240 mg/kg), as in the previous case, the highest decrease of acid phosphatase activity, compared to the control, was observed on day, while lowest on day 30 of the experiment. Addition of calcium peroxide caused an increase in pH of the soil (Table 3) and at the same time caused decrease of acid phosphatase activity in comparison with the activity of the enzyme observed in the control sample. By Bielińska (2005), optimum pH of the medium for acid phosphatase is in the range of 4–6. pH observed in this experiment was higher, what could affect on decrease of acid phosphatase activity. A similar phenomenon of acid phosphatase activity was observed by Quasenian et al. (2012), in studies of soil contaminated with anthracene, after 3 months of the experiment, they found a slight decrease in the activity of acid phosphatase.

The addition of fluoranthene in combination with calcium peroxide concentration of 120 mg/kg DW of the soil on day 1 of the experiment caused the highest percentage of inhibition of this enzyme. The effect of the activity of acid phosphatase was sinusoidal. No significant differences between the activity of acid phosphatase and the concentration of calcium peroxide were observed.

Statistical analysis of data indicated significant effects of the applied soil modifications only on day 1 of the experiment (the exception was the sample with the addition of fluoranthene) (Table 4).

**Table 4:** Changes of acid phosphatase activity during remediation of fluoranthene and statistical analysis of data

Soil samples	Acid phosphatase activity $\left[\frac{mg}{g\ast h}\right]$							
	1 day		7 days		14 days		30 days	
	AF ± SD	_S_	AF ± SD	_S_	AF ± SD	_S_	AF ± SD	_S_
Control sample	0.678 ± 0.004		0.510 ± 0.031		0.540 ± 0.071		0.425 ± 0.06	
1.5 mg fluoranthene	0.675 ± 0.008	−	0.473 ± 0.018	−	0.645 ± 0.027	+	0.553 ± 0.009	−
120 mg CaO_2_	0.427 ± 0.029	+	0.471 ± 0.046	−	0.463 ± 0.018	−	0.419 ± 0.013	−
240 mg CaO_2_	0.475 ± 0.004	+	0.477 ± 0.010	−	0.420 ± 0.018	+	0.422 ± 0.019	−
120 mg CaO_2_ + 1.5 mg fluoranthene	0.387 ± 0.01	+	0.466 ± 0.010	−	0.472 ± 0.015	−	0.413 ± 0.011	−
120 mg CaO_2_ + 1.5 mg fluoranthene	0.402 ± 0.018	+	0.452 ± 0.022	−	0.411 ± 0.007	+	0.421 ± 0.012	−

+ indicates that the difference in enzyme activity relative to the control was statistically significant at a 95 % confidence level. − indicates that the difference in enzyme activity relative to the control not statistically significant at a confidence level of 95 %

*AF* average acid phosphatase activity $\left[\frac{mg}{g\ast h}\right]$, *SD* standard deviation, *ts* significance of the differences between samples based on Student’s *t* test

### The Changes of Polyphenol Oxidase Activity During Remediation

Bacterial and fungal phenol oxidases are one of the most important enzymes involved in the degradation of polycyclic aromatic hydrocarbons (Baldrin et al. 2000; Chaudhary et al. 2012; Haritash and Kauskik 2009; Quasenian et al. 2011, 2012). These enzymes are released into the environment by secretion or cell lysis and are responsible for nonspecific catalysis of reactions such as oxidation of Mn^2+^ and Fe^2+^, polymerization, depolymerization, or transformation of a wide range of compounds. Due to the toxicity of the phenolic derivatives, the reactions catalyzed by phenol oxidases affect the activity and composition of the soil microbial population (Sinsabaugh 2010).

**Fig. 3:**
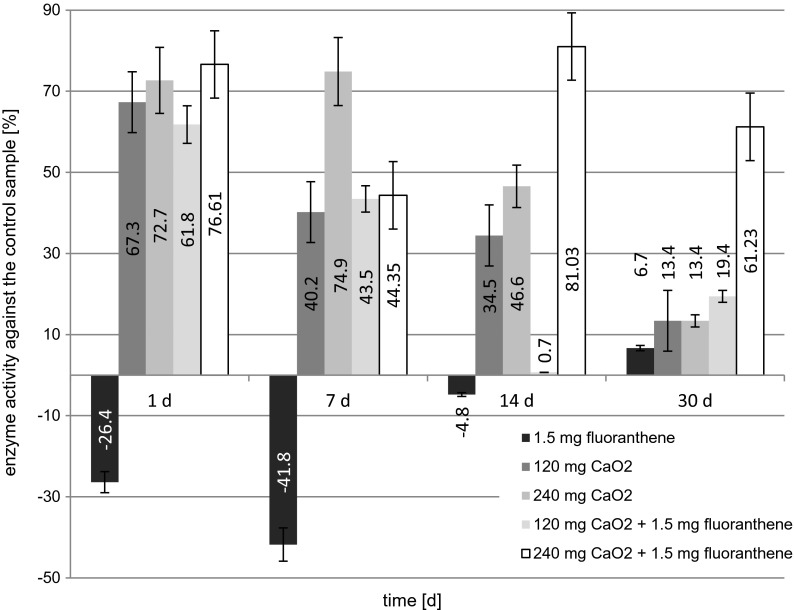
The effect of fluoranthene and calcium peroxide on the activity of polyphenol oxidase

The effect of the applied soil modifications on the activity of polyphenol oxidase activity is presented in Fig. 3. In the soil sample with fluoranthene, a stimulation of polyphenol oxidase was observed on days 1, 7, and 14 of the experiment, but on day 30, decrease of activity was observed compared to a control sample. The changes in the activity of this enzyme correlated with the changes of fluoranthene concentration in the soil sample. It was observed that the higher was the concentration of fluoranthene in a sample, the higher was the activity of polyphenol oxidase. This may suggest that polyphenol oxidase is actively participating in the degradation of polycyclic aromatic hydrocarbons and is immediately induced by soil microorganisms instantaneously when fluoranthene is introduced into the environment in an appropriate concentration. The same phenomena were observed by Patel et al. (2009), during the 15-day experiment of fluoranthene degradation. Acevado et al. (2011), on the contrary, found no phenol oxidase activity in a sample of soil containing polycyclic aromatic hydrocarbons.

The addition of calcium peroxide to soil samples, regardless of its concentration, caused the decrease of polyphenol oxidase activity during the whole experiment. Statistical analysis showed no significant difference between the results obtained for samples with the addition of 120 mg CaO_2_/kg DW of soil and 240 mg CaO_2_/kg DW of soil. The highest percentage decrease of phenol oxidase activity, amounting to 67.3 and 72.68 %, was recorded on day 1 of the study. Over time, the adverse effect of the addition of calcium peroxide on the activity of polyphenol oxidase decreased. In samples with the addition of fluoranthene and calcium peroxide, the percentage of decrease of polyphenol oxidase activity was similar as in the samples containing only calcium peroxide. Statistical analysis of results showed no significant difference between the samples with the addition of calcium peroxide and those contaminated with fluoranthene and with the addition of calcium peroxide.

Statistical analysis of the obtained results in comparison with a control sample is shown in Table 5. It was shown that all of the above modifications significantly affected the result of the test in comparison with a control sample. The exception was the sample contaminated with fluoranthene. In the course of the bioremediation process (day 14 and 30), there were no significant differences between the activity of polyphenol oxidase determined in the control sample and the sample contaminated with fluoranthene (Table 5). The decrease of polyphenol oxidase activity was probably due to the formation of highly reactive free hydroxyl radicals from calcium peroxide, which can inhibit the enzyme. However, different phenol oxidases were characterized by a different optimum pH. For example, the phenol oxidases of white rot fungi have the highest activity at pH 4–6, and the phenol oxidases of brown rot fungi have the highest activity at pH 6–7.5 (Courty et al. 2009). The positive correlation (3.52) between removal of fluoranthene and polyphenol oxidase activity has been observed; however, there was no correlation (1.085) in the case of acid phosphatase (for *p* = 0.05).

**Table 5:** Changes of polyphenol oxidase activity during remediation and statistical analysis of data

Soil samples	Acid phosphatase activity $\left[\frac{mg}{g\ast h}\right]$							
	1 day		7 days		14 days		30 days	
	AO ± SD	*t*_*S*_	AO ± SD	*t*_*S*_	AO ± SD	*t*_*S*_	AO ± SD	*t*_*S*_
Control sample	0.533 ± 0.016		0.324 ± 0.011		0.265 ± 0.045		0.491 ± 0.022	
1.5 mg fluoranthene	0.674 ± 0.100	+	0.459 ± 0.043	+	0.278 ± 0.035	−	0.458 ± 0.020	−
120 mg CaO_2_	0.174 ± 0.055	+	0.194 ± 0.027	+	0.174 ± 0.026	+	0.425 ± 0.015	+
240 mg CaO_2_	0.146 ± 0.024	+	0.081 ± 0.011	+	0.142 ± 0.028	+	0.425 ± 0.011	+
120 mg CaO_2_ + 1.5 mg fluoranthene	0.204 ± 0.036	+	0.183 ± 0.014	+	0.264 ± 0.014	−	0.395 ± 0.016	+
120 mg CaO_2_ + 1.5 mg fluoranthene	0.125 ± 0.017	+	0.180 ± 0.013	+	0.050 ± 0.012	+	0.190 ± 0.012	+

+ indicates that the difference in enzyme activity relative to the control was statistically significant at a 95 % confidence level. − indicates that the difference in enzyme activity relative to the control not statistically significant at a confidence level of 95 %

*AO* average polyphenol oxidase activity $\frac{\upmu \mathrm{mol}}{g\ast h}$, *SD* standard deviation, *ts* significance of the differences between samples based on Student’s *t* test

## Conclusions

The application of peroxides such as calcium peroxide allows shortening the duration of remediation of soils by increasing the content of oxygen available to the microorganisms and alkalinization of the environment. Addition of calcium peroxide to soil contaminated by fluoranthene caused approximately threefold increase in removal efficiency of this hydrocarbon on day 30 of bioremediation compared to the samples to which this reagent was not added. However, the increase of fluoranthene removal in the presence of calcium peroxide was observed, the slightly decrease of examined enzymes.

The presence of fluoranthene in soil stimulated phenol oxidase activity, which may indicate the important role of this enzyme in the decomposition of aromatic hydrocarbons.
